# Comparative Genome-Wide Alternative Splicing Analysis of Longissimus Dorsi Muscles Between Japanese Black (Wagyu) and Chinese Red Steppes Cattle

**DOI:** 10.3389/fvets.2021.634577

**Published:** 2021-04-29

**Authors:** Xibi Fang, Lixin Xia, Haibin Yu, Wei He, Zitong Bai, Lihong Qin, Ping Jiang, Yumin Zhao, Zhihui Zhao, Runjun Yang

**Affiliations:** ^1^College of Animal Science, Jilin University, Changchun, China; ^2^College of Coastal Agricultural Sciences, Guangdong Ocean University, Zhanjiang, China; ^3^Branch of Animal Husbandry, Jilin Academy of Agricultural Sciences, Changchun, China

**Keywords:** cattle, meat traits, fat deposition, muscle development, RNA-seq

## Abstract

Alternative splicing is a ubiquitous regulatory mechanism in gene expression that allows a single gene generating multiple messenger RNAs (mRNAs). Significant differences in fat deposition ability and meat quality traits have been reported between Japanese black cattle (Wagyu) and Chinese Red Steppes, which presented a unique model for analyzing the effects of transcriptional level on marbling fat in livestock. In previous studies, the differentially expressed genes (DGEs) in *longissimus dorsi muscle* (*LDM*) samples between Wagyu and other breeds of beef cattle have been reported. In this study, we further investigated the differences in alternative splicing in *LDM* between Wagyu and Chinese Red Steppes cattle. We identified several alternative splicing types including cassette exon, mutually exclusive exons, alternative 5′ splice site, alternative 3′ splice site, alternative start exon, and intron retention. In total, 115 differentially expressed alternatively spliced genes were obtained, of which 17 genes were enriched in the metabolic pathway. Among the 17 genes, 5 genes, including *MCAT, CPT1B, HADHB, SIRT2*, and *DGAT1*, appeared to be the novel spliced candidates that affect the lipid metabolism in cattle. Additionally, another 17 genes were enriched in the Gene Ontology (GO) terms related to muscle development, such as *NR4A1, UQCC2, YBX3/CSDA, ITGA7*, etc. Overall, altered splicing and expression levels of these novel candidates between Japanese black cattle and Chinese Red Steppes revealed by RNA-seq suggest their potential involvement in the muscle development and fat deposition of beef cattle.

## Introduction

Alternative splicing or alternative RNA splicing is a method to create different proteins from the same strand of DNA in mammalian cell. The structure and function of protein depend on the sequence of amino acids, which is dictated by the matured messenger RNA (mRNA). Current high-throughput sequencing technology suggests that alternative splicing is more widespread than initially thought, and alternative splicing is likely to be involved in various across tissue types and developmental stages as well as among individuals and populations (1). Hence, alternative splicing is an important and ubiquitous regulatory mechanism to increase the informational diversity and functional capacity of a single gene during posttranscriptional processing, and it also provide a crucial bridge between the genome and the proteome.

The currently accumulated data have highlighted importance of alternative splicing in normal physiology and pathology in human, including cancer, immune and infectious diseases, and metabolic conditions (2). With the development of requirements and technology, many studies used different methods to resolve the alternative splicing on a genome-wide scale (3, 4) including microarray (5), expressed sequence tag (EST) (6), and sequencing. Pan et al. (7) analyzed alternative splicing complexity in human tissues showing transcripts from ~95% of multiexon genes undergo alternative splicing by combining mRNA-Seq with expressed sequence tag (EST)-complementary DNA (cDNA) sequence data. The data of 15 diverse human tissue and cell line transcriptomes indicated that 92–94% of human genes undergo alternative splicing, and most alternative splicing and alternative cleavage events vary between tissues (8). These results also demonstrate the universality and utility of alternative splicing in mammal, and to further understand the alternative splicing will be a crucial effective method in the study of formation of human disease or perfect economic traits in livestock.

In previous studies, a larger number of transcriptomes were performed to analyze the genetics and to improve the production traits in livestock; the differentially expressed genes (DGEs) in *longissimus dorsi muscle* (*LDM*) samples among various breeds of beef cattle have been reported (9–12). However, the alternative splicing data on a genome-wide scale was rarely analyzed and studied in depth. In the present study, we analyzed the differences in expression levels and components of alternative splicing in *LMD* of Wagyu and Chinese Red Steppes cattle by RNA-seq, and the candidate functional alternative splicing types of genes related to meat traits were provided. The study allowed better genomic characterization of the cattle in terms of transcripts variability and analyzed the potential effect of alternative splicing of genes on muscle development and lipometabolism. It may provide a theoretical basis for the identification of functional transcripts and a potential genetic regulatory elements for the study of genetic mechanism of meat quality traits in beef cattle.

## Materials and Methods

### Ethics Statement

Animal care and experiments were performed according to the guidelines established by the care and use of laboratory animals of the Jilin University Animal Care and Use Committee (Approval ID: 20140310).

### Animal and Samples

The three Japanese black cattle (28 months old) were randomly chosen from 20 males born from cows that were artificially inseminated with semen stocks from the same bull. The three Chinese Red Steppes cattle (28 months old) were similarly chosen from 20 males with a common father. Three Japanese black cattle and three Chinese Red Steppes cattle were provided by the National Research Center for Animal Transgenic Bio-technology, Inner Mongolia University (Hohhot, China) and the Branch of Animal Science, Jilin Academy of Agricultural Sciences (Gongzhuling, China), respectively. The two farms housing the two groups of cattle were located at similar altitudes with similar natural weather conditions. The cattle in both groups were raised under similar normal conditions on a diet of corn and hay with access to feed and water *ad libitum*.

The marble scores of the 12–13 rib eye meat of these two breeds were accessed according to the classification of beef marbling grade standard. The marble scores of the three Wagyu cattle are 7, 7, and 6, which belong to the Grade 4 meat quality level (good grade). In the other group, the marble scores of the three Chinese Red Steppes cattle were 2, 2, and 2, respectively, which belonged to the Grade 2 meat quality level (below average grade). The tissue samples were transported in dry ice and stored in liquid nitrogen in the laboratory.

### RNA Extraction and Quality Analysis

Total RNA was isolated from *LDM* using Trizol reagent (Invitrogen, USA), treated with DNase I (NEB, Beijing, China), extracted with phenol-chloroform, and precipitated with ethanol. The quality and quantity of total RNA were determined using an Agilent 2100 Bioanalyzer (Agilent technologies, Palo Alto, CA). The mRNA was purified from total RNA using poly-T oligo-attached magnetic beads. From each sample, 1.5 μg of mRNA was used to construct six cDNA libraries for sequencing. The mRNA was treated with a Thermomixer (Eppendorf AG, Hamburg, Germany) to generate fragments with an average size of 200 bp (200 ± 25 bp) for the paired-end libraries. The fragmented mRNA was then used as templates for synthesizing the first-strand cDNA. The double-stranded cDNAs were purified and ligated to adaptors for Illumina paired-end sequencing. Library concentration was quantified by quantitative PCR (qPCR) and a Qubit® 2.0 Flurometer (Life Technologies, CA, USA), and the insert size was checked on an Agilent 2100 Bioanalyzer. The cDNA libraries were sequenced using the Illumina HiSeq2000 platform by the Beijing Genomics Institute, and 100 nt paired-end reads were generated. The raw data have been submitted to the Gene Expression Omnibus (GEO).

### Analysis of Alternative Splicing and Prediction of Novel Transcripts of Genes

Clean high-quality reads were obtained from the raw reads by removing the adaptor sequences and low-quality reads. An RNA-Seq TopHat (v2.0.6) splicing junction mapper was used for RNA-Seq alignment (13). We used our own localized reads to calculate the number of reads for each gene and the read per kilobase and read per million (RPKM) values. Other statistical results were also analyzed, such as gene range and depth and read distribution around start and stop codons.

The reads were mapped to the reference genome (*Bos taurus* UMD_3.1.1) using the ultra-fast, short-read mapping program Bowtie (http://www.bowtie-bio.sourceforge.net) (13). The mapped reads were assembled using MAQ (14) to identify possible splicing junctions, the unmapped reads are split into smaller segments to allow alignment to the reference genome, and splice junctions were defined by the seed expansion procedure. All junction reads must be at least 6 nt that exactly match each of the two adjacent areas of the potential junction sites, and all junction sites with N3 non-redundant reads in both group were filtered. Alternative splicing detector software based on junction reads (http://www.novelbio.com/asd/ASD.html) (15, 16), which recognizes and detects differential alternative splicing exons between two groups of RNA-Seq data, was used. We then counted the number of junction reads that matched either the inclusion or exclusion isoforms of both samples and calculated the *p*-value based on the junction read count between the samples using Fisher's exact test. Alternate exon read coverage for the corresponding gene was also calculated on the sample, and a second *p*-value based on the alternative exon read coverage for gene read coverage between samples was calculated using Fisher's exact test. The adjusted *p*-values were obtained by combining the two *p*-values using a weighted arithmetic expression to assess the statistical significance of alternative splicing between samples. Differentially expressed alternatively spliced genes between Japanese black cattle and Chinese Red Steppes were considered as differentially expressed according to adjusted *p* < 0.05.

### Gene Ontology and Kyoto Encyclopedia of Genes and Genomics Enrichment Analysis

Differentially expressed alternatively spliced genes were implemented by the Gene Ontology (GO) seq R package (17). GO was used to determine and compare the functions of the differentially expressed alternatively spliced genes as biological process, molecular function, and cellular component, with corrected *p* < 0.05 considered significantly enriched. Association of the genes with pathways was computed with the Kyoto Encyclopedia of Genes and Genomics (KEGG) (18–20). The pathway with a corrected *p* < 0.05 was considered as significantly enriched.

### Identification of Types and Quantification of Transcripts

One microgram total RNA was used to synthesize cDNA with the Transcriptor first-strand cDNA synthesis kit (Roche, USA) according to the manufacturer's recommendations. The primers were designed using Primer Premier 6.0 software (Premier BioSoft, Palo Alto, CA, USA) (Supplementary Tables 1, 2). The types of transcripts were identified by PCR in a 25.0 μl reaction volume including 12.5 μl of 2 × Taq PCR Master Mix (Vazyme, Nanjing, China), 0.5 μl of upstream and downstream primers (10 μM), 1.0 μl of cDNA, and 10.5 μl of ultrapure water. The PCR conditions were as follows: 95°C for 5 min; followed by 30 cycles of 95°C for 30 s, 57°C for 30 s, and 72°C for 1 min; and finally, 10 min extension at 72°C. The PCR products (8.0 μl) were run on 2% agarose gels.

Quantification of expression levels of differentially expressed alternatively spliced genes was detected by real-time PCR. Real-time PCR was performed using PCRmax Eco 48 (PCRmax, Staffordshire, UK). The real-time PCR system was as follows: 5 μl FastStart Universal SYBR Green Master (ROX) (Roche), 1 μl cDNA, 0.2 μl primer-F (10 μM), 0.2 μl primer-R (10 μM), and 3.6 μl RNase-free water. Reactions were incubated at 95°C for 10 min, followed by 40 cycles of 95°C for 10 s and 60°C for 30 s. β-Actin were used as reference genes, and relative expression levels were calculated using the 2^−ΔCt^. The data represent mean ± SEM from at least three independent experiments. Statistical analysis was performed by Student's *t* test at a significant level of *p* < 0.05.

## Results

### RNA-seq and Transcriptome Analysis of Chinese Red Steppes and Japanese Black Cattle in *LDM*

**Figure 1:**
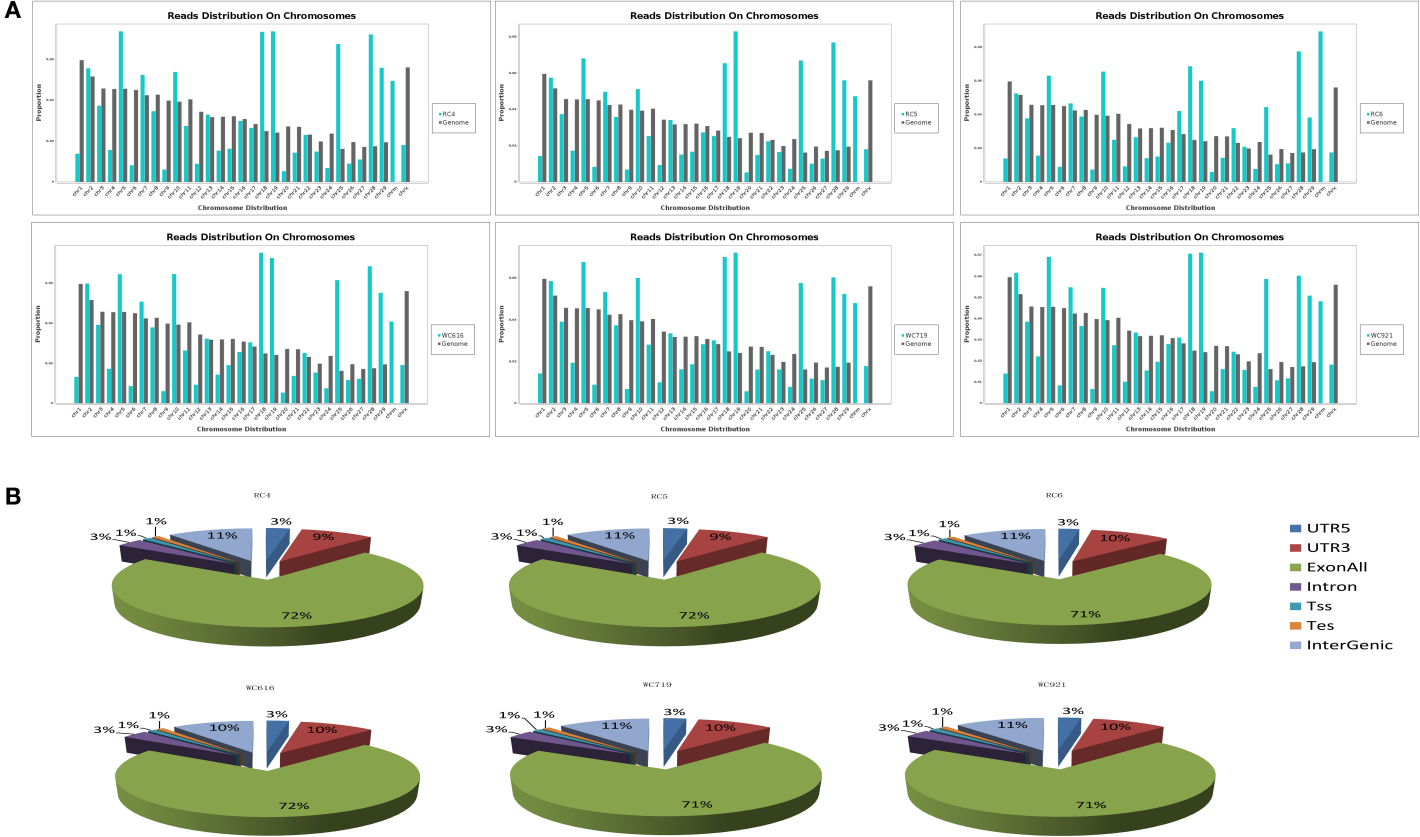
The average distribution coverage at chromosomes of mapped reads. **(A)** The average distribution coverage at different chromosomes of the genome about mapped reads. **(B)** Functional genomic elements distribution of messenger RNA (mRNA) reads in different groups.

A total of 268,490,636 reads were obtained from six samples in *LDM*. After filtering out low-quality data, 244,238,430 clean reads were mapped to *Bos taurus* (UMD_3.1.1). High-quality maps of the two breeds were obtained, and the unique mapping rates ranged from 87.3 to 88.3%. The details of the sequencing data quality of mRNA are shown in Table 1. The distribution of the reads on the chromosomes is shown in Figure 1A, and gene structure analysis was performed for each sample (Figure 1B). The sequence data were submitted to the GEO with the accession number of GSE161967.

**Table 1:** Details of sequencing data by RNA-seq.

**Samples**	**Total reads**	**Clean reads**	**UniqueMapped**	**ReadsFilter%**	**UniqueMappedRat%**
RC4	44,709,588	40,838,760	36,079,522	91.34	88.3
RC5	44,176,132	40,116,014	35,296,746	90.81	88.0
RC6	44,182,490	39,604,308	34,923,036	89.64	88.2
WC616	44,422,250	40,611,916	35,477,651	91.42	87.3
WC719	46,072,046	41,931,354	36,696,481	91.01	87.5
WC921	44,928,130	41,136,078	36,315,824	91.56	88.3

*WC616, WC719, WC921: longissimus dorsi muscle samples of Japanese black cattle (Wagyu); RC4, RC5, RC6: longissimus dorsi muscle samples of Chinese Red Steppes*.

### Forms and Expression of Alternative Splicing

**Figure 2:**
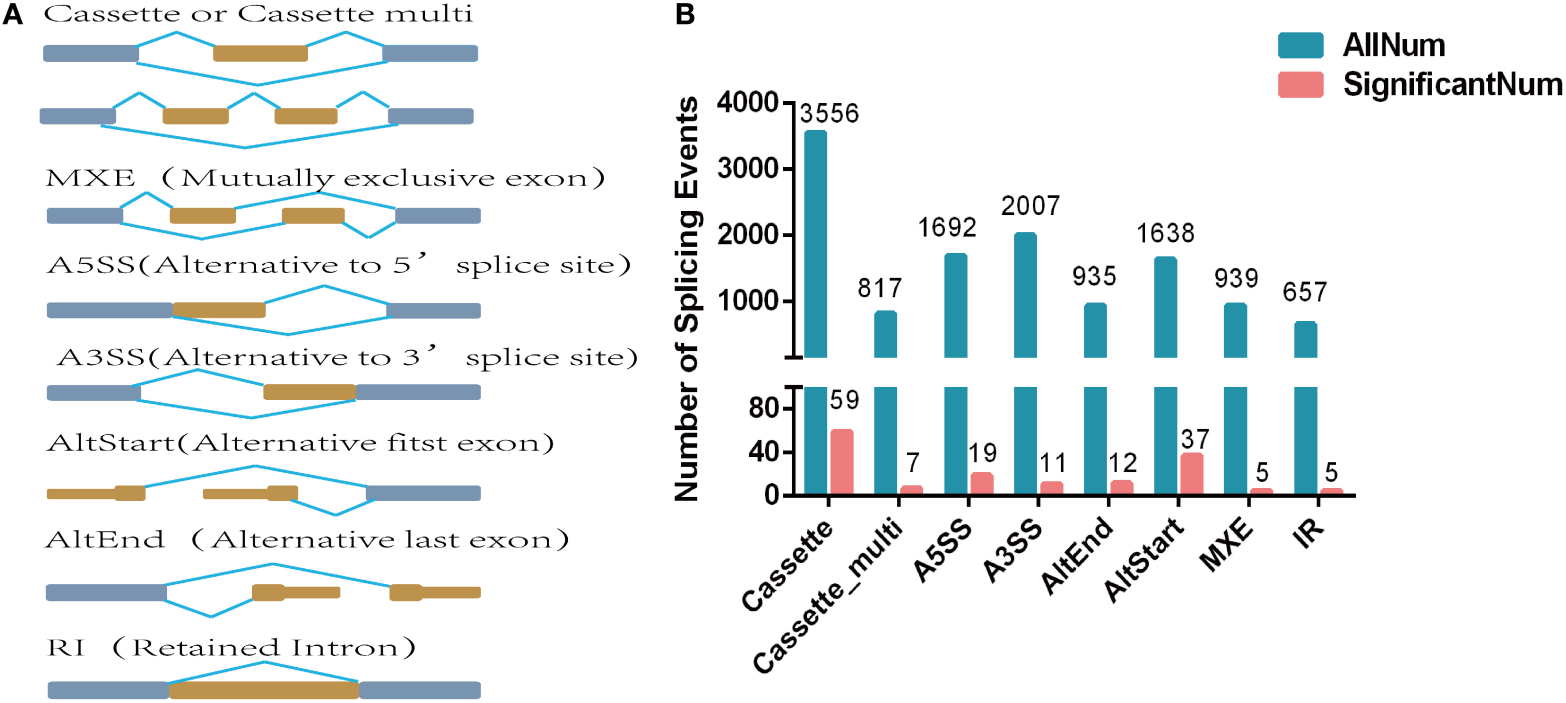
Differentially expressed alternatively spliced genes analysis. **(A)** Alternative splicing types. **(B)** Number of alternative splicing types. The blue blocks represent exons, and the yellow blocks represent introns.

A total of eight alternative splicing forms were obtained through data mapping analysis, including cassette exon (Cassette), cassette multiple exon (Cassette multi), alternative 5′ splice site (A5SS), alternative 3′ splice site (A3SS), alternative start exon (AltStart), alternative end exon (AltEnd), intron retention (IR), and mutually exclusive exons (Figure 2A). In the genome-wide analysis, 12,241 alternatively spliced events corresponding to 5,295 genes were identified. Cassette was the most abundant alternatively spliced types, followed by A3SS, A5SS, AltStart, MXE, AltEnd, Cassette multi, and IR.

A total of 155 differentially expressed alternatively spliced events between two breeds were screened according to adjusted *p* < 0.05. The differentially expressed alternatively spliced events refer to 59 Cassette, 7 Cassette multi, 11 A3SS, 19 A5SS, 12 AltEnd, 37 AltStart, 5 IR, and 5 MXE (Figure 2B). One hundred forty-four genes corresponding to differentially expressed alternative splicing were found between Chinese Red Steppes and Japanese black cattle including *TPM2, STRIP2, CPT1B, SYNPO2L, SIRT2*, etc. The top 15 differentially expressed alternatively spliced genes are listed in Table 2.

**Table 2:** Top 15 differentially expressed alternatively spliced genes between Japanese Black cattle and Chinese Red Steppes.

**AccID**	**Location**	**WCExp**	**RCExp**	**Adjusted_*p*-value**	**FDR**	**SplicingType**
TPM2	chr8:60271071-60271204	226999::101410	225046::98571	0	0	MXE
STRIP2	chr4:94155770-94155850	20::194	31::80	3.41E-08	2.09E-04	AltEnd
CPT1B	chr5:120103992-120104146	12::972	66::533	1.03E-07	4.19E-04	A5SS
SYNPO2L	chr28:29800955-29801849	158::930	44::778	2.46E-07	7.53E-04	Cassette
SIRT2	chr18:48879590-48879636	103::673	232::496	4.06E-07	9.95E-04	Cassette
ENSBTAG00000023039	chr28:31054477-31054529	534::678	688::708	1.02E-06	0.002084	Cassette
BOLA	chr23:28502996-28503043	13::752	309::552	1.42E-04	0.247944	Cassette
BCL2L1	chr13:61771313-61771393	43::264	107::199	1.86E-04	0.283899	AltStart
KBTBD12	chr22:60298315-60298395	167::908	116::780	2.34E-04	0.318568	AltStart
MLF1	chr1:109764134-109764196	447::744	595::736	3.03E-04	0.37118	Cassette
NDRG2	chr10:26137053-26137094	5861::5809	2570::3758	4.71E-04	0.523886	Cassette
NSUN2	chr20:66752104-66752177	165::177	305::178	5.47E-04	0.557585	IR
PKIG	chr13:73715532-73715612	289::185	252::147	7.78E-04	0.732468	AltStart
TNNT1	chr18:62726592-62726626	39418::33204	44061::32517	0.001065696	0.931799	Cassette
RBM24	chr23:39842778-39842890	102::427	104::325	0.001341344	1	Cassette

### GO and KEGG Enrichment of Differentially Expressed Alternatively Spliced Genes

**Figure 3:**
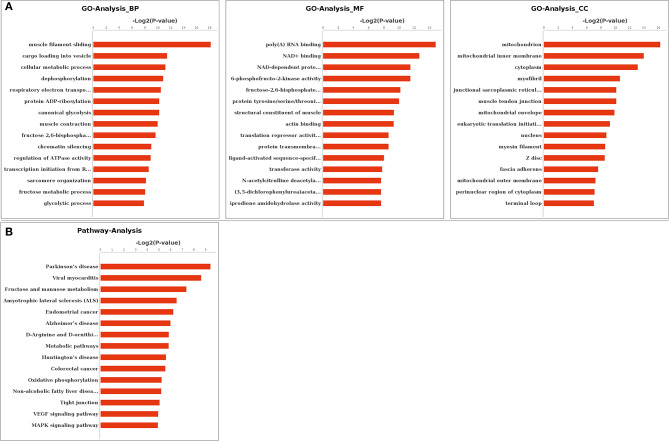
Gene Ontology (GO) and Kyoto Encyclopedia of Genes and Genomes (KEGG) enrichment. **(A)** GO enrichment of differentially expressed genes. **(B)** KEGG enrichment of differentially expressed genes. BP, biological processes; MF, molecular functions; CC, cellular components.

Functional enrichments on differentially expressed alternatively spliced genes by GO analysis found that 814, 532, and 232 GO terms were enriched in biological processes, molecular functions, and cellular components, respectively. A total of 216 biological processes, 115 molecular functions, and 54 cellular components were significantly enriched (*p* < 0.05). The most significantly enriched in biological processes included muscle filament sliding (GO: 0030049), muscle contraction (GO: 0006936), and cellular metabolic process (GO: 0044237). Muscle tendon junction (GO: 0005927), mitochondrion (GO: 0005739), and mitochondrial fatty acid beta-oxidation multienzyme complex (GO: 0016507) were among the most significantly enriched in cellular components. The most significantly enriched in molecular functions terms included poly(A) RNA binding (GO: 0044822), structural constituent of muscle (GO: 0008307), translation repressor activity, and nucleic acid binding (GO: 0000900). The top 15 GO terms in biological processes, molecular functions, and cellular components were listed by ascending order of corrected *p*-value, respectively (Figure 3A).

Functional enrichments on 59 differentially expressed alternatively spliced genes by KEGG analysis found that 138 pathways were enriched with 17 pathways significantly enriched (*p* < 0.05). These included metabolic pathway (PATH: 01100), vascular endothelial growth factor (VEGF) signaling pathway (PATH: 04370), mitogen-activated protein kinase (MAPK) signaling pathway (PATH: 04010), and apoptosis (PATH: 00730). The top 15 pathways are listed in Figure 3B by the ascending order of *p*-value.

### Identification of Alternative Splicing Types and Verification of Expression Levels of Transcripts

**Figure 4:**
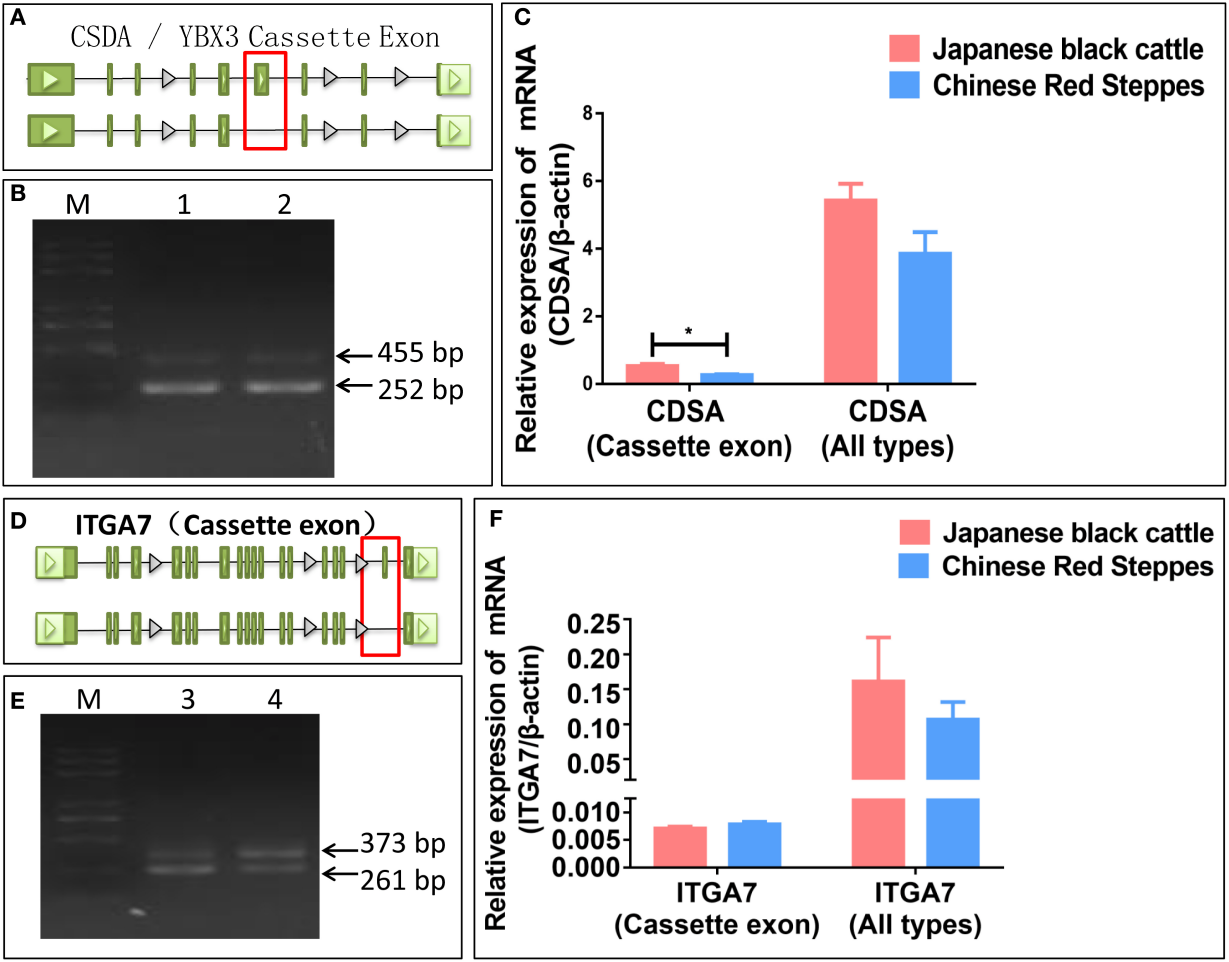
Verification of splicing type and expression level. **(A)** The alternative splicing pattern of CDSA. **(B)** PCR validation of CDSA (Cassette). **(C)** The relative expression levels of CDSA. **(D)** The alternative splicing pattern of ITGA7. **(E)** PCR validation of ITGA7 (Cassette). **(F)** The relative expression levels of ITGA7. (1) CDSA PCR product of *LDM* in Japanese black cattle, (2) CDSA PCR product of LDM in Chinese Red Steppes, (3) ITGA7 PCR product of *LDM* in Japanese black cattle, and (4) ITGA7 PCR product of LDM in Chinese red steppes (M) trans 2K Plus DNA Marker. Green blocks represent exons.

To further test the accuracy of our sequencing and prediction data, *integrin subunit alpha 7 (ITGA7)* and *Y-box binding protein 3 (YBX3/CSDA)* were validated by PCR and real-time PCR. *CSDA* was predicted and identified two transcripts and the long transcript with a cassette of exon (Figures 4A,B). The expression levels of the transcript with a cassette of exon is higher in Japanese black cattle, and the long transcript expression levels were significantly lower than short in two breeds (*p* < 0.05) (Figure 4C). *ITGA7* was also identified as having two transcripts, and the type of alternative splicing is cassette (Figures 4D,E); moreover, the expression levels of genes were higher in Japanese black cattle, and the long transcript with a cassette of exon was lower than in Chinese Red Steppes (Figure 4F). The results indicated that the alternative splicing prediction based on RNA-seq data was reliable.

## Discussion

In the present study, 12,241 alternatively spliced events corresponding to 5,295 genes indicate the ubiquity of alternatively spliced genes in the bovine genome, and cassette is the most abundant alternatively spliced types like other species of livestock; this distribution pattern is also similar to that of other animals reported previously (7, 21, 22). Comprehensively, these results suggest that animals might possess similar alternative splicing forms.

Numerous alternative splicing events occur during cell differentiation and tissue maturation, suggesting that alternative splicing supports proper development. Although the mechanisms and outcomes of alternative splicing of individual transcripts are relatively well-understood in dairy and beef cattle, such as *angiopoietin like 6 (ANGPTL6)* (23), *transmembrane protein 95 (TMEM95)* (24), and *calpain 3 (CAPN3)* (25), the scopes and exact functions of this regulatory mechanism still remain to be investigated on novel transcripts and alternative splicing networks.

According to the KEGG enrichment, a total of 59 alternatively spliced genes were enriched in 138 pathways, of which 17 genes including *phosphatidylinositol 4-kinase beta (PI4KB), diacylglycerol O-acyltransferase 1 (DGAT1), uridine-cytidine kinase 1 like 1 (UCKL1), NADH: ubiquinone oxidoreductase subunit C2 (NDUFC2)*, and *D-aspartate oxidase (DDO)* were enriched in metabolism pathways. The functional characterization of these genes is well-known in previous accumulated data. Therefore, we speculate that these genes also could mediate the formation of meat quality traits through the regulation of the related pathways by expression of different transcripts.

Although the differentially expressed alternative splicing in the same tissue of the same development stage should have totally similar alternative splicing, 155 differentially expressed alternatively spliced events between Japanese black cattle and Chinese Red Steppes were screened in the present study, which indicated that the corresponding genes or splicing events may be caused by the breed effect of fat deposition and muscle development capacity. Thus, understanding the function of the multiple transcripts and the effect of expression levels of each transcript such as *DGAT1, CPT1B, MCAT*, and *hydroxyacyl-CoA dehydrogenase trifunctional multi enzyme complex subunit beta (HADHB)* will be a key to study the formation of perfect quality meat traits in cattle.

The DNA sequence of *CPT1B* contains 8,755 bases, and it contains 19 exons and 18 introns reported in GenBank. *CPT1B* is reported as a key rate-limiting enzyme in the β-oxidation of fatty acids as well as playing an important role in regulating the decomposition and energy supply of fat (26, 27). Our previous study also found the single-nucleotide polymorphisms (SNPs) in the gene associated with meat quality. Moreover, the data of this study showed *CPT1B* has an alternative 5′ splice site at exon 7, and the novel transcript was a high expression in Japanese black cattle. Therefore, the result indicated that the expression of different transcripts of *CPT1B* between the two breeds may be related to the capability in fat deposition, while there is few report on alternative splicing of this gene; thus, the specific functions and mechanisms of the two transcripts need further verification.

*DGAT1* mediates triacylglycerol biosynthesis at the last committed step. It is widely known as the functional gene of milk fat and fatty acids contents (28–30), and also a gene could affect the fatty acids composition and fat content in the muscle and fat of beef cattle (31, 32). Alternative splicing of the *DGAT1* gene plays an important role in peanut triglyceride metabolism (33). Moreover, two alternative splicing types with different functions in triglyceride metabolism of *DGAT1* gene have been found in the diatom *Phaeodactylum tricornutum* (34). In the present study, two transcripts of *DGAT1* gene were also found in *LDM*; however, the structure and roles of alternative splicing are rarely reported in other studies in livestock; therefore, the functions of two alternative splicing types need further verification.

Although the present study found that many genes have novel transcripts with different expression levels, the specific genetic functions and regulation mechanisms of translated proteins by these mRNAs of the same gene on bovine muscle development, fat deposition, and meat quality traits still require further study.

## Conclusions

In this study, we disclosed features of genome-wide alternative splicing in two breeds with significant differences in fat deposition ability and meat quality traits through comprehensive transcriptome analysis by RNA-seq. The results suggest that 5,295 genes correspond to alternative splicing in bovine genome. Moreover, we found that cassette is the most abundant alternatively spliced types like other species. The analysis of functional categories demonstrates that differentially expressed alternatively spliced genes, including *MCAT, CPT1B, HADHB, SIRT2*, and *DGAT1*, have been reported to have regulatory effects on meat quality traits in previous studies. The results indicated that different types of alternative splicing and regulatory networks constructed by them in this study might partially contribute to the significant variance in meat quality traits between Japanese black and Chinese Red Steppes cattle. Therefore, the study provided a new direction for revealing the regulatory mechanism of gene regulatory elements on fat deposition and muscle development, which is the effects of different alternative splicing of candidate functional genes on marbling trait of beef cattle.

## Data Availability Statement

The datasets presented in this study can be found in online repositories. The names of the repository/repositories and accession number(s) can be found at: https://www.ncbi.nlm.nih.gov/genbank/, GSE161967.

## Ethics Statement

The animal study was reviewed and approved by laboratory animals of the Jilin University Animal Care and Use Committee (Approval ID: 20140310).

## Author Contributions

ZZ and RY designed the study. LQ and PJ collected the samples. XF and HY collected the data. XF, LX, ZB, and WH conducted the bioinformatics analyses and wrote the manuscript. WH and YZ revised the manuscript. All authors read and approved the final manuscript.

## Conflict of Interest

The authors declare that the research was conducted in the absence of any commercial or financial relationships that could be construed as a potential conflict of interest.

## Abbreviations

MCAT: malonyl-CoA-acyl carrier protein transacylase
CPT1B: carnitine palmitoyltransferase 1B
HADHB: hydroxyacyl-CoA dehydrogenase trifunctional multi enzyme complex subunit β
SIRT2: sirtuin 2
DGAT1: diacylglycerol O-acyltransferase 1
NR4A1: nuclear receptor subfamily 4 group A member 1
UQCC2: ubiquinol-cytochrome c reductase complex assembly factor 2
YBX3/CSDA: Y-box binding protein 3
ITGA7: integrin subunit alpha 7.
