# Supplementary material for: Comparative Genome-Wide Alternative Splicing Analysis of Longissimus Dorsi Muscles Between Japanese Black (Wagyu) and Chinese Red Steppes Cattle
Source: Front Vet Sci. 2021 Apr 29;8:634577. doi: 10.3389/fvets.2021.634577 (PMC8116494; doi:10.3389/fvets.2021.634577)
Supplement: Supplementary file 1 [file Data_Sheet_1.docx]

Supplementary Material

# Supplementary Tables

**Table S1. The identify primers for different alternatively spliced types**

| Gene name | Sequence ID | Primer name | Primer sequces (5’ to 3’) | length (bp) |
| --- | --- | --- | --- | --- |
| ITGA7 | 506953 | ITGA7(AS)s | ACTTGCTGCTCAGAGATGCT | 261/373 |
|  |  | ITGA7(AS)as | CCGTCTTCTCCTCCTTGAACT |  |
| CSDA | 533297 | CSDA(AS)S | GTACATCAGACTGCCATCAAGA | 455/252 |
|  |  | CSDA(AS)as | CCATCCTTCATCTCACCAATCT |  |

**Table S2. The real-time PCR primers of differentially expressed alternatively spliced genes**

| Gene name | Sequence ID | Primer name | Primer sequces (5’ to 3’) |
| --- | --- | --- | --- |
| CSDA | NM_001113531.1 | CSDA(AS)RT S | CGTGGGACAGACCTTTGAC |
|  |  | CSDA(AS)RT AS | GCGGTAAGTCGGATTCCTATG |
|  |  | CSDA(AS)RT 1S | CGGTGAGATTGGTGAGATGAAG |
|  |  | CSDA(AS)RT 1AS | GCCGCTTGTTGATTCTCCTTAT |
| ITGA7 | NM_001191305.1 | ITGA7(AS)RT S | AGTGTGGCTTCTTCCGTCG |
|  |  | ITGA7(AS)RT AS | CCGTCTTCTCCTCCTTGAACT |
|  |  | ITGA7(AS)RT 1S | GCCTCTGGAACAGCACCTT |
|  |  | ITGA7(AS)RT 1AS | AGCATCTCTGAGCAGCAAGT |
